# Laparoscopic enteropexy for prolapsing ileostomy

**DOI:** 10.1308/003588412X13373405386015m

**Published:** 2012-09

**Authors:** T Papettas, L Wong

**Affiliations:** University Hospital Coventry and Warwickshire NHS Trust,UK

Stomal prolapse is a complication caused by invagination of proximal redundant bowel through the stoma. We describe a laparoscopic technique for repairing a prolapsing end ileostomy that confers the benefits of being minimally invasive and preserves the existing stoma site.

Three laparoscopic ports are inserted in the standard way: umbilical (12mm), left hypochondrial (12mm) and left iliac fossa (5mm). After laparoscopic inspection, the prolapsing small bowel is reduced appropriately and positioned against the abdominal wall to perform the enteropexy. The small bowel mesentery is then sutured to the abdominal wall using interrupted polypropylene. This method is effective and avoids the risks of fistula formation.

**Figure 1:**
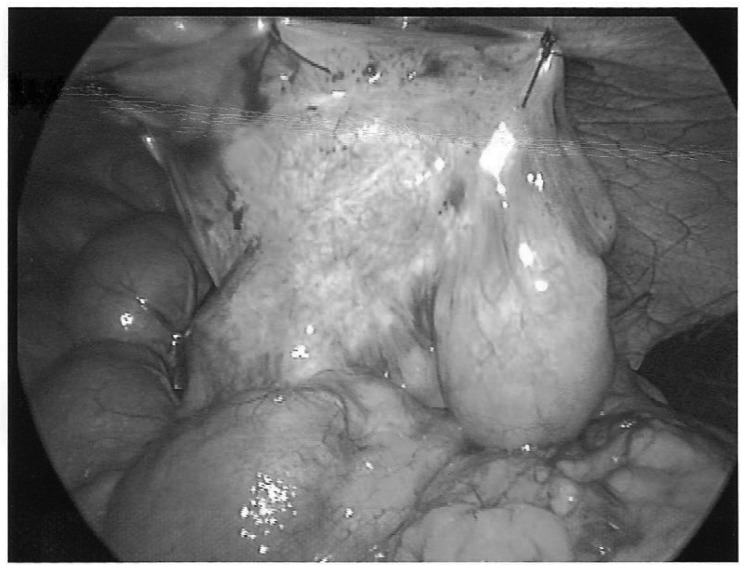
Enteropexy performed by suturing intussuscepting thickened small bowel mesentery to the abdominal wall

